# Cultures and cures: neurodiversity and brain organoids

**DOI:** 10.1186/s12910-021-00627-1

**Published:** 2021-05-17

**Authors:** Andrew J. Barnhart, Kris Dierickx

**Affiliations:** Department of Public Health and Primary Care, Centre for Biomedical Ethics and Law, KU Leuven, Leuven, Belgium

**Keywords:** Brain organoids, Autism, Neurodiversity, Disability, Research, Bioethics

## Abstract

**Background:**

Research with cerebral organoids is beginning to make significant progress in understanding the etiology of autism spectrum disorder (ASD). Brain organoid models can be grown from the cells of donors with ASD. Researchers can explore the genetic, developmental, and other factors that may give rise to the varieties of autism. Researchers could study all of these factors together with brain organoids grown from cells originating from ASD individuals. This makes brain organoids unique from other forms of ASD research. They are like a multi-tool, one with significant versatility for the scope of ASD research and clinical applications. There is hope that brain organoids could one day be used for precision medicine, like developing tailored ASD drug treatments.

**Main body:**

Brain organoid researchers often incorporate the medical model of disability when researching the origins of ASD, especially when the research has the specific aim of potentially finding tailored clinical treatments for ASD individuals. The neurodiversity movement—a developmental disability movement and paradigm that understands autism as a form of natural human diversity—will potentially disagree with approaches or aims of cerebral organoid research on ASD. Neurodiversity advocates incorporate a social model of disability into their movement, which focuses more on the social, attitudinal, and environmental barriers rather than biophysical or psychological deficits. Therefore, a potential conflict may arise between these perspectives on how to proceed with cerebral organoid research regarding neurodevelopmental conditions, especially ASD.

**Conclusions:**

Here, we present these perspectives and give at least three initial recommendations to achieve a more holistic and inclusive approach to cerebral organoid research on ASD. These three initial starting points can build bridges between researchers and the neurodiversity movement. First, neurodiverse individuals should be included as co-creators in both the scientific process and research communication. Second, clinicians and neurodiverse communities should have open and respectful communication. Finally, we suggest a continual reconceptualization of illness, impairment, disability, behavior, and person.

## Background

Following the rapid evolution in stem cell scientific advancements, brain organoids are beginning to impact neuroscience significantly. These “mini-brains,” about 4mm in diameter, can be grown from cells taken from patients and used for many kinds of neurobiological research. Already, brain organoids have been used to discover neurological and developmental mechanisms underlying connections between the Zika virus and infants born with microcephaly [1]. Organoid models have helped bring successful advancements in understanding other neurological conditions such as Parkinson’s disease, Alzheimer’s disease, and traumatic brain injury [2, 3]. Research with brain organoids is now making strides toward understanding the origins of neurodevelopmental conditions like Autism Spectrum Disorder (ASD). Researchers can grow ASD brain organoid models with the cells of ASD patients and explore the genetic, developmental, and other factors that may give rise to the varieties of autism. In addition, brain organoids can be used to test different pharmaceuticals. With the rise of individually-tailored clinical treatments, some have speculated that individually-tailored drugs and other therapies could be on the horizon for ASD patients. Brain organoids made from their (induced pluripotent or adult) stem cells can be used to test particular drugs in a safe environment, or else CRISPR/Cas9 could be used to test altering genes in the brain organoid and develop targeted gene therapies [4]. All of this could bring significant improvements to the clinical outcomes for patients with ASD.

Yet as this biotechnology further progresses in application and development, tensions between the biomedical research community and various disability communities could increase. At first glance, it may be difficult to imagine how anyone would oppose research that could be used to effectively treat or cure neurodevelopmental conditions. The scope, use, and uniqueness of brain organoids in ASD research and clinical applications place them squarely into a biotechnological category with a promising innovative future. But, for those in what is known as the neurodiversity movement, using brain organoids to help find treatments or even cures for conditions like ASD is precisely the problem. Considering the neurodiversity perspective, the grounding of neurological conditions like ASD in brain organoids could potentially contradict the pluralistic social value of diversity itself.

## Main text

### Organoids and modeling disability

Research fields on ASD have ranged from genetics, epigenetics, neurology, and developmental biology, to name a few. But what makes brain organoid research so different is that brain organoids are a unique multi-tool, one that has significant versatility in terms of the scope of autism research and clinical applications. The ASD research fields listed above can all be studied—together—with brain organoids. There is a wide array of ASD studies utilizing brain organoids for etiology [5], genetics [4, 6], epigenetics [7], developmental biology [8], and drug discovery [9]. And as previously mentioned, there are significant hopes that the results of these brain organoid studies will one day be used for individualized tailored treatments and regenerative medicine.

In a way, we can break down the multi-tool of brain organoids into a couple of categories. On the one hand, brain organoids are utilized as models representing either the brain more generally or the brain of an individual patient. Brain organoids seek to model neurodevelopmental conditions like ASD by explaining the etiology of perceived behavioral and communication disorders in terms of neurological, genetic, or environmental factors. By using brain organoids, scientists are now pointing to FOXG1 transcription factors [6], mutations in the TRPC6 gene [10], alterations in DLX6 gene expression [9], and many other relevant factors that may influence the diverse forms of ASD.

On the other hand, testing and creating new pharmaceuticals and clinical treatments with brain organoid models is beginning to take shape. Some examples include gene-therapies and pharmaceuticals tested *in vitro* on brain organoids [9–11]. While experiments with brain organoids for ASD are in their infancy, these examples show the large potential clinical impact of brain organoid models.

In all of these ways, brain organoids are therefore often used as a medical model of disability. Under the medical model, disability originates from a physical, psychological, or functional impairment within the individual. Such impairments are, in turn, primarily the result of disease, deficits, defects, or disorders—an abnormality [12, 13]. For ASD, the model contents that there is a deficit or disorder in the genetics or neurological development within the individual, leading to the various psychological impairments associated with the condition, which in turn leads to the behavioral disability of autism. This model of thinking can be directly seen in at least some of the ASD brain organoid research. For example, Ilieva et al. describe ASD as “characterized by deficits in social cognition and communication as well as behavioral inflexibility” [14]. Yang and Shcheglovitov propose a “precision medicine pipeline for diagnostic and drug-discovery for ASD” by deriving “patient-specific organoids to screen for cellular and molecular deficits in individuals with idiopathic ASDs” [15]. Choi et al. use the phrases “Make a Healthy Organoid Autistic” and “Make Autistic Organoids Healthy” in two flow diagrams that depict growing patient-derived organoids and altering them with CRISPR/Cas9 techniques [11]. Incidentally, the depictions of the autism organoids are shown as dark brown, almost decaying, while the healthy organoids are depicted with a glowing, seemingly pure, white light emanating from within. The flow diagrams present autism as unhealthy and something to be avoided, while not having autism is the best possible scenario.

Furthermore, from the medical model, such impairments and disorders need to be treated, rehabilitated, or cured since they are also seen as primary sources of social exclusion for disabled people, including ASD individuals [12, 13, 16]. In a neurodevelopmental disability like ASD, treatment or cure would occur by discovering new personalized drugs or other therapies. The hope is that with brain organoids modeling ASD in a dish, research on new drugs could be conducted in a safe, non-invasive environment. Combining new personalized pharmacological and behavioral treatments could lead to better clinical outcomes for patients with ASD [10].

The medical model of disability has undoubtedly improved the lives of many people through medical intervention. It shows that what people with disabilities experience can be rooted in something real and identifiable, physically or psychologically. With organoids, for example, personalized treatments for individuals with cystic fibrosis have made significant progress as intestinal organoids model the condition in a more realistic, human way [17]. The model, however, suffers from what Nesse and Stein suggest, “a temptation to conceptualize disorders in an essentialist way that oversimplifies reality” [18]. In other words, there is very little room for the social contexts that may influence the well-being of the individual. Indeed, a chief criticism from proponents of other disability models is that the medical model often ignores the daily social and environmental contexts which influence the experiences of disabled people.

### Neurodiversity

For many disability advocates, autism is not a pathology, disorder, or deficit. Autism is a difference and a form of human diversity that is worthy of value. Neurodiversity is a conceptual paradigm and disability movement that invites us to conceive autistic neurology and other neurodevelopmental differences as contributing to overall human neurological and cultural diversity [19]. It is often seen as a response to the medical model of autism, originating as an online grassroots movement in the late 1990s and the term initially coined by sociologist Judy Singer [20, 21]. The movement celebrates autism as an integral part of individual identity and as something that should not be altered [22]. Its adherents oppose the pathologization of autism and incorporate the social model of disability into the conceptual framework [20, 22, 23]. The underlying idea of the social model is that disability arises from social, attitudinal, and environmental barriers rather than from the impairments themselves [16].

According to Walker, there are three axioms to the neurodiversity paradigm, which are paraphrased as follows [20, 24]: (1) Autism is a form of human diversity and is worthy of value. (2) A “normal” or “healthy” mind or brain are social and cultural constructions and are in a similar category as a “normal” or “right” ethnicity, gender, or culture. (3) The social dynamics—including those of power, inequality, and creative potential—of neurodiversity are similar to other forms of human diversity. Because of these axioms, the neurodiversity movement contains political and cultural contexts, as those within the movement often respond to social oppression faced by neurodiverse people [25].

However, the weaknesses of the neurodiversity perspective shouldn’t be discounted. One problem stems from understanding which forms of neurological differences count as constituents of neurodiversity [26]. For instance, should people with Down syndrome, Parkinson’s, Timothy syndrome, and so on be counted as neurodiverse constituents? Would neurological difference alone be enough to be counted as a neurodiverse individual? Even in reference only to autism (as some neurodiversity proponents may prefer), the problem of constituency may still arise when considering distinctions between “high-functioning” and “low-functioning” autism. As an example, some people with Asperger’s may consider it worse stigmatization by being placed in the same neurological constituency with those who are considered low-functioning [26]. On the other hand, other autism advocates have rejected this high and low functioning distinction either because there is little genetic basis for the distinction or because the distinction may devalue low-functioning autistic individuals.

Constituency problems aside, the neurodiversity movement does offer considerable strengths. Its key strengths include the ability to point out misconceptions surrounding the capabilities of people with autism and its ability to expand upon fundamental concepts like functioning, normalcy, natural variation, and even diversity as applied to autism. By situating these strengths into the social model of disability, the neurodiversity movement counts itself as an essential part in the struggle for disability civil rights.

### Brain organoids and diversity

Western pluralistic societies indeed state that they value different forms of diversity. Such societies value different ethnicities, religions, languages, genders, and sexualities for various reasons. Since these forms and behaviors of human diversity are valued, any research conducted that may impact these differences should be done in a respectful and inclusive fashion. Additionally, scientific research that explores valued forms of diversity should be scrutinized. As an example, scientific studies have not found the “gay gene” [27, 28]. But scientists could continue searching for the gene(s) using brain organoids, finding a more concrete neurological basis for sexuality and orientation, and maybe even the ability to detect and alter it. On the other hand, as LGBTQ+ communities would probably argue, such research is not at all important, and scientists should avoid medicalizing sexual behavior in this manner. If researchers do consider continuing with a search for the gay gene(s) via brain organoid techniques, then they should remember the lessons from previously including LGBTQ+ communities into their research practices.

The neurodiversity movement puts forth these same propositions for autism and other neurodevelopmental differences. If a society values diversity, then that society should value neurodiversity in much the same way as other forms. To value diversity is to respect and include the wide array of people and their differences. To value neurodiversity, then, would be the same as to accept other forms of human variation. The aim is to value people with autism as “*fully persons* rather than as broken beings in need of repair” [29]. The neurodiversity movement calls for a more inclusive discussion in scientific research in order to communicate findings in a mutually respectful way [30]. No doubt it would call for the same degree of inclusion and consideration given to relevant communities for scientific research in sexuality and sexual orientation. There is even a common disability activist saying for this request, *nothing about us without us*, and researchers who study brain organoids would do well to consider it.

Moreover, the neurodiversity movement calls for pluralistic societies to rethink the very reasons behind exploring the underlying genetics and biology of neurodevelopmental differences. To help, scientists should reflect on at least a few questions. Should the knowledge gained from brain organoids be used in ways that may minimize atypical behaviors and neurodiverse people in society? Or should the knowledge be used in ways that may remove social, attitudinal, and environmental barriers for neurodiverse people? How should people who consider themselves neurodiverse be brought into the scientific analysis and discussion surrounding brain organoids? And how should a pluralistic society, one that truly values diversity in all of its forms, answer these questions? Such questions are, of course, difficult to answer. Therefore, a few initial recommendations toward answering them are necessary.

First, researchers can strive for the inclusion and integration of neurodiverse people into the various research stages, as previously mentioned. It might be said that such inclusion is unrealistic or too difficult. The *nothing about us, without us* call to action could be countered with the truism *if you’ve met one person with autism, you’ve met one person with autism*. That is to say, the diversity within the autism spectrum is such that any representative from an autism constituency could never actually, accurately, or fully represent the autism community itself, thus rendering the whole exercise moot. We counter this by pointing once again to the recent genetic research on sexual orientation and the inclusion efforts towards the LGBTQ+ community. It is fair to say that the LGBTQ+ community is diverse and often considers sexuality and sexual orientation as a spectrum. It can be said here, too, that if you’ve met one LGBTQ+ person, you’ve met one LGBTQ+ person. While individuals do provide their own perspective, this does not negate the sense of communal identity individuals often share. The conflict of individual versus communal perspectives should not prevent researchers from including them in the research process; otherwise, they may risk creating further tensions towards already vulnerable communities. While imperfect, it is better to have some perspectives of a diverse community than having none altogether out of a concern over the inability to represent every single member of that community. Moreover, there is the point that autism and neurodiversity advocates have themselves been asking for more co-creative inclusion in the scientific process, especially with research that relates to autism [31].

A potential consequence of this initial step of bringing neurodiverse stakeholders into the science of brain organoids for autism is to potentially change the principle aims of this research. By incorporating a more neurodiverse perspective, there may be a shift of focus by using brain organoids to better understand the phenomenological elements or the experiences of autism rather than the etiological. Should this occur and, as Anderson and Cushing have suggested, there were to be an increased understanding of the phenomenological aspects of autism, it may then be seen as having a value in-of-itself [32].

An essential second step is understanding that this call for more stakeholder inclusion in frontier research with brain organoids should not be mistaken as a call for stifling biomedical professionals from saying anything negative about particular cognitive conditions. As Goering suggests, such professionals should still be able to speak candidly about the negatives of conditions yet still maintain respect for certain positions that neurodiversity advocates advance [33]. There are justified reasons why clinical professionals want their patients with autism to seek particular treatments and why patients with autism want to seek these treatments. This understanding of speaking candidly, honestly, and with mutual respect is a necessary part of the next step.

A final potential starting point is to continue reconsidering the distinctions between illness, impairment, disability, behavior, and person. With regard to reconsidering and reconceiving autism, a more moderate position within the neurodiversity movement is building a conceptual middle ground between a view which pathologizes autism and a social constructivist view [23]. The more radical position within the neurodiversity movement would be to advocate for the outright rejection of considering autism in terms of deficit or disorder and potentially push for a more firm social constructivist view [23]. The neurodiversity movement would do well to consider how other models of disability (e.g., International Classification of Functioning, Nagi Model, or the Capabilities Approach) might be incorporated into their movement [16]. Indeed, this is an important aim for the moderate position within the movement [23].

As an example, the capabilities approach to neurodiversity begins with incorporating human variation as the “starting point for justice,” in addition to first looking at what resources are available to individuals [34]. These available resources combine with the personal characteristics and one’s (social, physical, economic, cultural, and political) environment to create practical opportunities for individuals. From these practical opportunities, a person makes suitable choices for themselves so as to achieve their own well-being [16]. Brain organoid research, and the clinical applications that result from it, may be seen as an available resource for neurodiverse people. It can be one avenue to maximize the practical opportunities for neurodiverse individuals.

Furthermore, it may be necessary to include neurodiverse individuals as co-creators in the scientific process to maximize practical opportunity. But exploring the intersections of neurodiversity, the capabilities approach, and brain organoid research is beyond the scope of this work. We only intend to raise awareness of possible tensions going forward between ASD brain organoid research and neurodiversity. However, such an intersection may be a worthwhile starting point to ease or avoid these tensions.

## Conclusions

Advancements in neuroscience should not solely be seen as a threat to minimize people with neurodevelopmental differences. Researchers are making remarkable strides with brain organoids in enhancing our understanding of people with disabilities, and people as a whole. However, researchers should be mindful of conflating disease with impairment or disability and consider how best to communicate their ideas with respect and care. If we truly value biomedicine and what it can do for our health, the forward strides in brain organoid research should not be unnecessarily hindered. And if we truly value diversity, and believe that neurodiversity is worthy of value, then we need to deeply consider the potential impacts frontier research with brain organoids could have on these values.

## Data Availability

Not applicable.
